# Risk factors for ICD-10-coded Respiratory Syncytial Virus-associated deaths in hospitalized patients in Germany before the COVID-19 pandemic (nationwide in-patient data, 2010–2019)

**DOI:** 10.1007/s15010-025-02712-8

**Published:** 2025-12-16

**Authors:** Patricia Niekler, David Goettler, Johannes Liese, Andrea Streng

**Affiliations:** Department of Pediatrics, University Hospital Würzburg, Josef-Schneider-Straße 2, 97080 Würzburg, Germany

**Keywords:** Respiratory syncytial virus, Hospitalization, Risk factors, Severe outcome, In-hospital fatality

## Abstract

**Purpose:**

We compared nationwide data on the clinical characteristics of deceased and non-deceased patients with Respiratory Syncytial Virus (RSV)-coded hospitalization to evaluate potential risk factors for in-hospital fatality by age group.

**Methods:**

Data from International Statistical Classification of Diseases (10th Revision)-based German Hospital Statistics for patients from 2010–2019 with a primary discharge diagnosis code for RSV-related pneumonia (J12.1), bronchitis (J20.5) or bronchiolitis (J21.0) were assessed by remote data retrieval. Selected underlying conditions and complications were reported stratified by age group and outcome.

**Results:**

Overall, 612 (0.3% of 205,352) RSV-coded patients died in hospital (103 children < 18 years, 51 adults 18–59 years, 458 seniors > 59 years). Children and adults with underlying chronic cardiovascular, neurological, immunological, or lower respiratory diseases had a higher risk of dying than those without (Odds Ratio 109, 58, 28, 6 in children, and 3, 3, 3, 2 in adults). In seniors, the risk was increased for patients with chronic neurological conditions (OR 1.3) but not for other underlying conditions. Acute respiratory distress syndrome, sepsis and pneumonia increased the risk of a fatal outcome in all age groups.

**Conclusion:**

In-hospital fatality of RSV-coded patients varied considerably with age, chronic conditions and complications. Seniors were the most affected age group and may therefore benefit from the RSV vaccination recommended in Germany since 2024 for all over 75 years and seniors with pre-existing conditions.

## Introduction

Severe lower respiratory tract infections (LRTI) by respiratory syncytial virus (RSV) mostly affect young children. Older persons, especially seniors and/or those with underlying diseases, can also become severely ill, with substantial RSV-associated mortality rates particularly among those ≥ 75 years [1–5]. However, severe infections and fatalities in adults and seniors are likely underreported, mainly due to the lack of systematic testing for viral respiratory pathogens especially before the COVID-19 pandemic. Clinical data on fatal RSV infections is limited [1–5].

Previously, we had described the clinical characteristics of all 205,352 patients in Germany hospitalized for RSV-LRTI during the period 2010–2019, with an RSV-LRTI code according to the International Statistical Classification of Diseases and Related Health Problems (10th Revision), German Modification (ICD-10) [6]. During this period, a total of 612 in-hospital fatalities were reported (in-hospital fatality rate: 0.3%), of which 103 (0.05%) were children, 51 (3.9%) adults, and 458 (7.8%) seniors, and with the in-hospital fatality rate increasing continuously with age (from 0.02% in children < 1 year of age to 14.3% in seniors ≥ 90 years) [6].

The aim of the present analyses is to compare the clinical characteristics of the deceased and non-deceased hospitalized RSV-LRTI patients in order to evaluate potential risk factors for in-hospital fatality by age.

## Methods

We conducted a retrospective, observational study of RSV-coded hospitalizations using a remote data query on fully anonymized patient datasets recorded in the German Hospital Statistics database of the German Federal Statistical Office [6]. Annual reporting of patient key information to this database is mandatory for all hospitals in Germany. The main data query included all in-patients from the years 2010–2019 with a primary ICD-10 diagnosis code for RSV pneumonia (J12.1), RSV bronchitis (J20.5) or RSV bronchiolitis (J21.0) at discharge; in supplementary analyses, patients with any RSV code were considered [6]. Based on the ICD-10-documented secondary diagnoses, selected chronic diseases (immune disorders, adiposity, diabetes, chronic diseases of the lower respiratory tract/circulatory system/nervous system, and—in children—congenital/perinatal disorders) and complications (bacterial pneumonia, sepsis, acute respiratory distress syndrome) were reported. In the present analyses, data were presented stratified by age group (children < 18 years, adults 18–59 years, and seniors > 59 years) and outcome (non-deceased/deceased). Differences between subgroups (significance p < 0.05) were reported using Chi^2^/Fisher’s Exact test and odds ratio (OR; deceased *vs* non deceased) with 95% confidence interval (95%CI).

## Results

Analyses were performed on 103 deceased and 198,036 non-deceased children, 51 deceased and 1262 non-deceased adults, and 458 deceased and 5442 non-deceased seniors.

In the 103 deceased children (89% < 5 years), the most frequently reported underlying diseases were diseases of the cardiovascular and nervous system (52% and 42%, including congenital manifestations; Table 1). Children with underlying cardiovascular/neurological/immune/lower respiratory tract disease had a higher risk of dying (OR 109/58/28/6; all p < 0.05) than children without these diseases; in children with congenital/perinatal conditions, this risk was especially high for those with nervous system malformation/lung disease/Down syndrome (OR 34/26/18; all p < 0.01).

**Table 1:** Characteristics of non-deceased (n = 204,740) and deceased (n = 612) patients with RSV-coded hospitalization (primary diagnosis), by age group (Data from German Statistical Office, January 2010-December 2019)

Age group (years)												
	< 18				18–59				> 59			
Patients with RSV-LRTI^a^	All	Non-deceased	Deceased	OR (95%CI), p-value	All	Non-deceased	Deceased	OR (95%CI), p-value	All	Non-deceased	Deceased	OR (95%CI), p-value
	N = 198,139	N = 198,036	N = 103		N = 1313	N = 1262	N = 51		N = 5900	N = 5442	N = 458	
Male	111,593 (56.3)	111,546 (56.3)	47 (45.6)	0.7 (0.4; 1.0)*	712 (54.2)	681 (54.0)	31 (60.8)	1.3 (0.8; 2.4)	2581 (43.7)	2367 (43.5)	214 (46.7)	1.1 (0.9; 1.4)
Underlying chronic conditions^b^												
Immune disorder (C00–C99, B20–B24, D8–D9, Z94)	597 (0.3)	589 (0.3)	8 (7.8)	28.2 (13.7; 58.4)**	469 (35.7)	438 (34.7)	31 (60.8)	2.9 (1.6; 5.2)**	841 (14.3)	780 (14.3)	61 (13.3)	0.9 (0.7; 1.2)
Chronic disease of lower respiratory tract (without asthma) (J40-J44, J47)	1841 (0.9)	1836 (0.9)	5 (4.9)	5.5 (2.2; 13.4)*	232 (17.7)	217 (17.2)	15 (29.4)	2.0 (1.1; 3.7)*	1210 (20.5)	1104 (20.3)	106 (23.1)	1.2 (0.9; 1.5)
Bronchial asthma (J45–J46)	1341 (0.7)	1341 (0.7)	0 (–)	–	69 (5.3)	XXX	XXX	–	204 (3.5)	196 (3.6)	8 (1.7)	0.5 (0.2; 1.0)*
Disease of circulatory system (I00–I99)	2038 (1.0)	1984 (1.0)	54 (52.4)	108.9 (73.8; 160.7)**	552 (42.0)	517 (41.0)	35 (68.6)	3.2 (1.7; 5.8)**	4894 (82.9)	4509 (82.9)	385 (84.1)	1.1 (0.8; 1.4)
Disease of nervous system (G00–G99)	2468 (1.2)	2425 (1.2)	43 (41.7)	57.8 (39.0; 85.7)**	245 (18.7)	227 (18.0)	18 (35.3)	2.5 (1.4; 4.5)*	1287 (21.8)	1170 (21.5)	117 (25.5)	1.3 (1.0; 1.6)*
Adiposity (E66)	104 (0.1)	104 (0.1)	0 (–)	–	99 (7.5)	96 (7.6)	3 (5.9)	0.8 (0.2; 2.5)	291 (4.9)	273 (5.0)	18 (3.9)	0.8 (0.5; 1.3)
Diabetes (E10–E14)	32 (0.0)	32 (0.02)	0 (–)	–	188 (4.3)	181 (14.3)	7 (13.7)	1.0 (0.4; 2.1)	1766 (29.9)	1623 (29.8)	143 (31.2)	1.1 (0.9; 1.3)

Patients are reported as n (% of N). XXX = censored due to low patient number (n < 3). Odds ratio (OR) with 95% confidence interval (95%CI) between deceased vs non-deceased

p-values from Chi^2^-/Fisher’s Exact test; significance level: *p < 0.05, **p < 0.01

^a^Respiratory Syncytial Virus—Lower Respiratory Tract Infection, defined by ICD-10; RSV pneumonia J12.1, RSV bronchitis J20.5, RSV bronchiolitis J21.0

^b^In children, congenital disorders of the organ systems were associated with fatal outcome (all p < 0.01): nervous (n = 776 all; 764 non-deceased, 12 deceased), OR 34, 95%CI 19–62 (ICD-10 Q00–Q07); circulatory (n = 2945; 2929, 16), OR 12, 95%CI 7–21 (Q20–Q28); respiratory (n = 543; 540, 3), OR 11, 95%CI 3–35 (Q30–Q34); Down syndrome (n = 1041; 1032, 9), OR 18, 95%CI 9–36 (Q90); chronic respiratory originating in the perinatal period (n = 468; 462, 6), OR 26 95%CI 12–61 (P27)

In the 51 adults who died (65% 50–59 years), underlying cardiovascular/immune disorders were most frequent (69%/61%) and adults with these diseases had the highest risk for in-hospital fatality (OR 3/3), followed by adults with disorders of the nervous system (OR 2.5) or the lower respiratory tract (OR 2) (all p < 0.05).

Of 458 deceased seniors, 89% were ≥ 70 years. Most frequent were chronic cardiovascular diseases/diabetes/nervous system diseases (84%/31%/26%). Patients with nervous system disorders had an increased risk of dying (OR 1.3, p < 0.05), in contrast to patients without such disorders.

For children/adults/seniors, the risk for in-hospital fatality increased in case of RSV-associated pneumonia (OR 10/17/5), acute respiratory distress syndrome (OR 749/17/9), sepsis (OR 110/17/6) and bacterial pneumonia (OR 8/7/2), with all p < 0.05 (Table 2).

**Table 2:** Diagnoses/complications (selection) of non-deceased (n = 204,740) and deceased (n = 612) patients with RSV-coded hospitalization (primary diagnosis), by age group (Data from German Statistical Office, January 2010–December 2019)

Age group (years)												
	< 18				18–59				> 59			
Patients with RSV-LRTI^a^	All	Non-deceased	Deceased	OR (95%CI), p-value	All	Non-deceased	Deceased	OR (95%CI), p-value	All	Non-deceased	Deceased	OR (95%CI), p-value
	N = 198,139	N = 198,036	N = 103	–	N = 1313	N = 1262	N = 51	–	N = 5900	N = 5442	N = 458	
RSV pneumonia (J12.1)	60,972 (30.8)	60,888 (30.7)	84 (81.6)	10.0 (6.1; 16.4)*	793 (60.4)	744 (59.0)	49 (96.1)	17.1 (4.1; 70.5)**	3450 (58.5)	3058 (56.2)	392 (85.6)	4.6 (3.6; 6.0)**
RSV bronchitis (J20.5)	62,402 (31.5)	62,394 (31.5)	8 (7.8)	0.2 (0.1; 0.4)**	470 (35.8)	467 (37.0)	3 (5.9)	0.11 (0.03; 0.3)**	2293 (38.9)	2225 (40.9)	68 (14.8)	0.3 (0.2; 0.3)**
RSV bronchiolitis (J21.0)	79,080 (39.9)	79,068 (39.9)	12 (11.7)	0.2 (0.1; 0.4)**	63 (4.8)	63 (5.0)	0 (–)	–	193 (3.3)	190 (3.5)	3 (0.7)	0.2 (0.1; 0.6)**
Bacterial pneumonia (J13–J18)	3853 (2.0)	3938 (2.0)	15 (14.6)	8.4 (4.9; 14.5)**	138 (10.5)	117 (9.3)	21 (41.2)	6.9 (3.8; 12.4)**	421 (7.1)	358 (6.6)	63 (13.8)	2.3 (1.7; 3.0)**
Sepsis (A40–A41)	272 (0.1)	259 (0.1)	13 (12.6)	110.0 (60.9; 199.8)**	58 (4.4)	40 (3.2)	18 (35.3)	16.7 (8.7; 32.1)**	216 (3.7)	147 (2.7)	69 (15.1)	6.4 (4.7; 8.7)**
ARDS (J80)	74 (0.1)	56 (0.03)	18 (17.5)	748.7 (422.5; 1326.5)**	28 (2.1)	18 (1.4)	10 (19.6)	16.9 (7.3; 38.8)**	41 (0.7)	24 (0.4)	17 (3.7)	8.7 (4.6; 16.3)**

Patients are reported as n (% of N). Odds ratio (OR) with 95% confidence interval (95%CI) between deceased vs non-deceased

*ARDS* acute respiratory distress syndrome

p-values from Chi^2^-/Fisher’s Exact test; significance level: *p < 0.05, **p < 0.01

^a^Respiratory Syncytial Virus—Lower Respiratory Tract Infection, defined by ICD-10; RSV pneumonia J12.1, RSV bronchitis J20.5, RSV bronchiolitis J21.0

## Discussion

Of the documented RSV-coded in-hospital deaths from 2010–2019, 75% were in seniors ≥ 60 years; with an in-hospital fatality rate of 7.8%. These percentages were similar to those of in-hospital influenza-associated fatalities during the same period (87% in seniors; in-hospital fatality rate 6.4%) [7], in line with other studies [1, 3, 5, 8]. The lower absolute numbers of reported RSV fatalities in seniors compared to influenza fatalities (458 vs 4700) may partly result from RSV underdiagnosis, as testing for RSV in seniors in case of respiratory symptoms was usually not performed, most likely due to the lack of an RSV-specific therapy.

Overall, chronic conditions known as risk factors for RSV-associated hospitalizations also increased the risk for in-hospital fatality in children and adults [2], especially immune disorders, and underlying diseases of the circulatory and nervous system. Interestingly, deceased seniors with RSV-LRTI had a slightly higher rate of neurological diseases than non-deceased, but showed similar rates for most other types of chronic conditions, indicating that older age in itself, associated with immunosenescence, is a relevant risk factor for RSV-related mortality [2]. RSV-associated pneumonia and severe complications were associated with fatal outcome in all three age groups.

Among other limitations [6], our present comparison of deceased and non-deceased RSV-LRTI patients probably underestimated RSV-associated fatalities as we included only patients with RSV-LRTI coded as primary diagnosis (representing the main reason for the hospital admission). Previous, supplementary analyses with extended search strategies to identify RSV-coded patients showed that if patients were included with any primary diagnosis referring to the respiratory system in combination with any secondary RSV code, the overall number of fatalities was 1.5 times higher (n = 928); if patients with any RSV code were included in the search strategy, the overall number of fatalities was 2.8 times higher (n = 1732), but may have included patients with ‘incidental’ RSV unrelated to the hospital stay [6].

However, the main factor for underestimating possible RSV involvement especially in older age groups in the years before the COVID-19 pandemic most probably was underdiagnosis due to the lack of hospital admission tests on RSV in patients with respiratory symptoms. In the post-pandemic era, such tests are now more common (e.g., in the form of ‘triple tests’ on influenza, SARS-CoV-2 and RSV) and may thus allow a re-evaluation of the burden of RSV-hospitalizations and fatalities in adults and seniors in future years. Since August 2024, RSV vaccination is recommended in Germany for all ≥ 75 years, and for those ≥ 60 years with severe underlying disease. A more accurate estimate of the burden of severe RSV disease especially in adults and seniors could allow for assessing a potential positive impact of the vaccination even for those groups not covered by the current recommendation, for example adults with and seniors without underlying diseases.

In conclusion, our data show that in-hospital fatality of RSV-coded patients varied considerably with age and underlying conditions. Seniors, especially those ≥ 70 years, were most affected and, hence, could therefore benefit from regular hospital admission tests for RSV in case of respiratory symptoms, and especially from the recently recommended RSV vaccination. However, underdiagnosis of RSV-associated hospitalizations in adults and seniors in the pre-pandemic years is likely and requires reassessment in the post-pandemic era where hospital admission testing for respiratory viruses occurs more frequently.

## Data Availability

Data Source: Research Data Center (RDC) of the Federal Statistical Office and Statistical Offices of the Länder (Germany), DRG-Statistik 2010–2019, 10.21242/23141.2010.00.00.1.1.0 to 10.21242/23141.2019.00.00.1.1.0; own calculations (project 4458-2021). The analysis programs and the resultant data extractions are not publicly available.
